# Characteristics and prognosis of bloodstream infection in patients with COVID-19 admitted in the ICU: an ancillary study of the COVID-ICU study

**DOI:** 10.1186/s13613-021-00971-w

**Published:** 2021-12-24

**Authors:** Nicolas Massart, Virginie Maxime, Pierre Fillatre, Keyvan Razazi, Alexis Ferré, Pierre Moine, Francois Legay, Guillaume Voiriot, Marlene Amara, Francesca Santi, Saad Nseir, Stephanie Marque-Juillet, Rania Bounab, Nicolas Barbarot, Fabrice Bruneel, Charles-Edouard Luyt, the COVID ICU Bacteremia Study Group on behalf of the COVID-ICU Investigators, Alain Mercat, Pierre Asfar, François Beloncle, Julien Demiselle, Tài Pham, Arthur Pavot, Xavier Monnet, Christian Richard, Alexandre Demoule, Martin Dres, Julien Mayaux, Alexandra Beurton, Cédric Daubin, Richard Descamps, Aurélie Joret, Damien Du Cheyron, Frédéric Pene, Jean-Daniel Chiche, Mathieu Jozwiak, Paul Jaubert, Guillaume Voiriot, Muriel Fartoukh, Marion Teulier, Clarisse Blayau, Laetitia Bodenes, Nicolas Ferriere, Johann Auchabie, Anthony Le Meur, Sylvain Pignal, Thierry Mazzoni, Jean-Pierre Quenot, Pascal Andreu, Jean-Baptiste Roudau, Marie Labruyère, Saad Nseir, Sébastien Preau, Julien Poissy, Daniel Mathieu, Sarah Benhamida, Rémi Paulet, Nicolas Roucaud, Martial Thyrault, Florence Daviet, Sami Hraiech, Gabriel Parzy, Aude Sylvestre, Sébastien Jochmans, Anne-Laure Bouilland, Mehran Monchi, Marc Danguy des Déserts, Quentin Mathais, Gwendoline Rager, Pierre Pasquier, Jean Reignier, Amélie Seguin, Charlotte Garret, Emmanuel Canet, Jean Dellamonica, Clément Saccheri, Romain Lombardi, Yanis Kouchit, Sophie Jacquier, Armelle Mathonnet, Mai-Ahn Nay, Isabelle Runge, Frédéric Martino, Laure Flurin, Amélie Rolle, Michel Carles, Rémi Coudroy, Arnaud W. Thille, Jean-Pierre Frat, Maeva Rodriguez, Pascal Beuret, Audrey Tientcheu, Arthur Vincent, Florian Michelin, Fabienne Tamion, Dorothée Carpentier, Déborah Boyer, Christophe Girault, Valérie Gissot, Stéphan Ehrmann, Charlotte Salmon Gandonniere, Djlali Elaroussi, Agathe Delbove, Yannick Fedun, Julien Huntzinger, Eddy Lebas, Grâce Kisoka, Céline Grégoire, Stella Marchetta, Bernard Lambermont, Laurent Argaud, Thomas Baudry, Pierre-Jean Bertrand, Auguste Dargent, Christophe Guitton, Nicolas Chudeau, Mickaël Landais, Cédric Darreau, Alexis Ferre, Antoine Gros, Guillaume Lacave, Fabrice Bruneel, Mathilde Neuville, Jérôme Devaquet, Guillaume Tachon, Richard Gallo, Riad Chelha, Arnaud Galbois, Anne Jallot, Ludivine Chalumeau Lemoine, Khaldoun Kuteifan, Valentin Pointurier, Louise-Marie Jandeaux, Joy Mootien, Charles Damoisel, Benjamin Sztrymf, Matthieu Schmidt, Alain Combes, Juliette Chommeloux, Charles Edouard Luyt, Frédérique Schortgen, Leon Rusel, Camille Jung, Florent Gobert, Damien Vimpere, Lionel Lamhaut, Bertrand Sauneuf, Liliane Charrrier, Julien Calus, Isabelle Desmeules, Benoît Painvin, Jean-Marc Tadie, Vincent Castelain, Baptiste Michard, Jean-Etienne Herbrecht, Mathieu Baldacini, Nicolas Weiss, Sophie Demeret, Clémence Marois, Benjamin Rohaut, Pierre-Henri Moury, Anne-Charlotte Savida, Emmanuel Couadau, Mathieu Série, Nica Alexandru, Cédric Bruel, Candice Fontaine, Sonia Garrigou, Juliette Courtiade Mahler, Maxime Leclerc, Michel Ramakers, Pierre Garçon, Nicole Massou, Ly Van Vong, Juliane Sen, Nolwenn Lucas, Franck Chemouni, Annabelle Stoclin, Alexandre Avenel, Henri Faure, Angélie Gentilhomme, Sylvie Ricome, Paul Abraham, Céline Monard, Julien Textoris, Thomas Rimmele, Florent Montini, Gabriel Lejour, Thierry Lazard, Isabelle Etienney, Younes Kerroumi, Claire Dupuis, Marine Bereiziat, Elisabeth Coupez, François Thouy, Clément Hoffmann, Nicolas Donat, Anne Chrisment, Rose-Marie Blot, Antoine Kimmoun, Audrey Jacquot, Matthieu Mattei, Bruno Levy, Ramin Ravan, Loïc Dopeux, Jean-Mathias Liteaudon, Delphine Roux, Brice Rey, Radu Anghel, Deborah Schenesse, Vincent Gevrey, Jermy Castanera, Philippe Petua, Benjamin Madeux, Otto Hartman, Michael Piagnerelli, Anne Joosten, Cinderella Noel, Patrick Biston, Thibaut Noel, Gurvan L. E. Bouar, Messabi Boukhanza, Elsa Demarest, Marie-France Bajolet, Nathanaël Charrier, Audrey Quenet, Cécile Zylberfajn, Nicolas Dufour, Buno Mégarbane, Sébastian Voicu, Nicolas Deye, Isabelle Malissin, François Legay, Matthieu Debarre, Nicolas Barbarot, Pierre Fillatre, Bertrand Delord, Thomas Laterrade, Tahar Saghi, Wilfried Pujol, Pierre Julien Cungi, Pierre Esnault, Mickael Cardinale, Vivien Hong Tuan Ha, Grégory Fleury, Marie-Ange Brou, Daniel Zafimahazo, David Tran-Van, Patrick Avargues, Lisa Carenco, Nicolas Robin, Alexandre Ouali, Lucie Houdou, Christophe Le Terrier, Noémie Suh, Steve Primmaz, Jérome Pugin, Emmanuel Weiss, Tobias Gauss, Jean-Denis Moyer, Catherine Paugam Burtz, Béatrice La Combe, Rolland Smonig, Jade Violleau, Pauline Cailliez, Jonathan Chelly, Antoine Marchalot, Cécile Saladin, Christelle Bigot, Pierre-Marie Fayolle, Jules Fatséas, Amr Ibrahim, Dabor Resiere, Rabih Hage, Clémentine Cholet, Marie Cantier, Pierre Trouiler, Philippe Montravers, Brice Lortat-Jacob, Sebastien Tanaka, Alexy Tran Dinh, Jacques Duranteau, Anatole Harrois, Guillaume Dubreuil, Marie Werner, Anne Godier, Sophie Hamada, Diane Zlotnik, Hélène Nougue, Armand Mekontso-Dessap, Guillaume Carteaux, Keyvan Razazi, Nicolas De Prost, Nicolas Mongardon, Meriam Lamraoui, Claire Alessandri, Quentin de Roux, Charles de Roquetaillade, Benjamin G. Chousterman, Alexandre Mebazaa, Etienne Gayat, Marc Garnier, Emmanuel Pardo, Lea Satre-Buisson, Christophe Gutton, Elise Yvin, Clémence Marcault, Elie Azoulay, Michael Darmon, Hafid Ait Oufella, Geoffroy Hariri, Tomas Urbina, Sandie Mazerand, Nicholas Heming, Francesca Santi, Pierre Moine, Djillali Annane, Adrien Bouglé, Edris Omar, Aymeric Lancelot, Emmanuelle Begot, Gaétan Plantefeve, Damien Contou, Hervé Mentec, Olivier Pajot, Stanislas Faguer, Olivier Cointault, Laurence Lavayssiere, Marie-Béatrice Nogier, Matthieu Jamme, Claire Pichereau, Jan Hayon, Hervé Outin, François Dépret, Maxime Coutrot, Maité Chaussard, Lucie Guillemet, Pierre Goffin, Romain Thouny, Julien Guntz, Laurent Jadot, Romain Persichini, Vanessa Jean-Michel, Hugues Georges, Thomas Caulier, Gaël Pradel, Marie-Hélène Hausermann, Thi My Hue Nguyen-Valat, Michel Boudinaud, Emmanuel Vivier, Sylvène Rosseli, Gaël Bourdin, Christian Pommier, Marc Vinclair, Simon Poignant, Sandrine Mons, Wulfran Bougouin, Franklin Bruna, Quentin Maestraggi, Christian Roth, Laurent Bitker, François Dhelft, Justine Bonnet-Chateau, Mathilde Filippelli, Tristan Morichau-Beauchant, Stéphane Thierry, Charlotte Le Roy, Mélanie Saint Jouan, Bruno Goncalves, Aurélien Mazeraud, Matthieu Daniel, Tarek Sharshar, Cyril Cadoz, Rostane Gaci, Sébastien Gette, Guillaune Louis, Sophe-Caroline Sacleux, Marie-Amélie Ordan, Aurélie Cravoisy, Marie Conrad, Guilhem Courte, Sébastien Gibot, Younès Benzidi, Claudia Casella, Laurent Serpin, Jean-Lou Setti, Marie-Catherine Besse, Anna Bourreau, Jérôme Pillot, Caroline Rivera, Camille Vinclair, Marie-Aline Robaux, Chloé Achino, Marie-Charlotte Delignette, Tessa Mazard, Frédéric Aubrun, Bruno Bouchet, Aurélien Frérou, Laura Muller, Charlotte Quentin, Samuel Degoul, Xavier Stihle, Claude Sumian, Nicoletta Bergero, Bernard Lanaspre, Hervé Quintard, Eve Marie Maiziere, Pierre-Yves Egreteau, Guillaume Leloup, Florin Berteau, Marjolaine Cottrel, Marie Bouteloup, Matthieu Jeannot, Quentin Blanc, Julien Saison, Isabelle Geneau, Romaric Grenot, Abdel Ouchike, Pascal Hazera, Anne-Lyse Masse, Suela Demiri, Corinne Vezinet, Elodie Baron, Deborah Benchetrit, Antoine Monsel, Grégoire Trebbia, Emmanuelle Schaack, Raphaël Lepecq, Mathieu Bobet, Christophe Vinsonneau, Thibault Dekeyser, Quentin Delforge, Imen Rahmani, Bérengère Vivet, Jonathan Paillot, Lucie Hierle, Claire Chaignat, Sarah Valette, Benoït Her, Jennifier Brunet, Mathieu Page, Fabienne Boiste, Anthony Collin, Florent Bavozet, Aude Garin, Mohamed Dlala, Kais Mhamdi, Bassem Beilouny, Alexandra Lavalard, Severine Perez, Benoit Veber, Pierre-Gildas Guitard, Philippe Gouin, Anna Lamacz, Fabienne Plouvier, Bertrand P. Delaborde, Aïssa Kherchache, Amina Chaalal, Jean-Damien Ricard, Marc Amouretti, Santiago Freita-Ramos, Damien Roux, Jean-Michel Constantin, Mona Assefi, Marine Lecore, Agathe Selves, Florian Prevost, Christian Lamer, Ruiying Shi, Lyes Knani, Sébastien Pili Floury, Lucie Vettoretti, Michael Levy, Lucile Marsac, Stéphane Dauger, Sophie Guilmin-Crépon, Hadrien Winiszewski, Gael Piton, Thibaud Soumagne, Gilles Capellier, Jean-Baptiste Putegnat, Frédérique Bayle, Maya Perrou, Ghyslaine Thao, Guillaume Géri, Cyril Charron, Xavier Repessé, Antoine Vieillard-Baron, Mathieu Guilbart, Pierre-Alexandre Roger, Sébastien Hinard, Pierre-Yves Macq, Kevin Chaulier, Sylvie Goutte, Patrick Chillet, Anaïs Pitta, Barbara Darjent, Amandine Bruneau, Sigismond Lasocki, Maxime Leger, Soizic Gergaud, Pierre Lemarie, Nicolas Terzi, Carole Schwebel, Anaïs Dartevel, Louis-Marie Galerneau, Jean-Luc Diehl, Caroline Hauw-Berlemont, Nicolas Péron, Emmanuel Guérot, Abolfazl Mohebbi Amoli, Michel Benhamou, Jean-Pierre Deyme, Olivier Andremont, Diane Lena, Julien Cady, Arnaud Causeret, Arnaud De La Chapelle, Christophe Cracco, Stéphane Rouleau, David Schnell, Camille Foucault, Cécile Lory, Thibault Chapelle, Vincent Bruckert, Julie Garcia, Abdlazize Sahraoui, Nathalie Abbosh, Caroline Bornstain, Pierre Pernet, Florent Poirson, Ahmed Pasem, Philippe Karoubi, Virginie Poupinel, Caroline Gauthier, François Bouniol, Philippe Feuchere, Anne Heron, Serge Carreira, Malo Emery, Anne Sophie Le Floch, Luana Giovannangeli, Nicolas Herzog, Christophe Giacardi, Thibaut Baudic, Chloé Thill, Said Lebbah, Jessica Palmyre, Florence Tubach, David Hajage, Nicolas Bonnet, Nathan Ebstein, Stéphane Gaudry, Yves Cohen, Julie Noublanche, Olivier Lesieur, Arnaud Sément, Isabel Roca-Cerezo, Michel Pascal, Nesrine Sma, Gwenhaël Colin, Jean-Claude Lacherade, Gauthier Bionz, Natacha Maquigneau, Pierre Bouzat, Michel Durand, Marie-Christine Hérault, Jean-Francois Payen

**Affiliations:** 1 Service de Réanimation, CH de St BRIEUC, 10, rue Marcel Proust, 22000 Saint-Brieuc, France; 2 Surgical and Medical Intensive Care Unit Hôpital, Raymond Poincaré, 9230 Garches, France; 3 AP-HP, Hôpitaux Universitaires Henri-Mondor, Service de Médecine Intensive Réanimation, 94010 Créteil, France; 4 Univ Paris Est Créteil, INSERM, IMRB, 94010 Créteil, France; 5 Université Paris Est Créteil, Faculté de Médecine de Créteil, IMRB, GRC CARMAS, 94010 Créteil, France; 6 Service de Réanimation/USC, Hôpital Mignot, Centre hospitalier de Versailles, 177 rue de Versailles, 78150 Le Chesnay, France; 7 Service de Médecine Intensive Réanimation, Hôpital Tenon, Assistance Publique-Hôpitaux de Paris, Sorbonne Université, and Groupe de Recherche Clinique CARMAS, Collegium Galilée, Créteil, France; 8 Centre de Réanimation, CHU de Lille, 59000 Lille, France; 9 INSERM U1285, Université de Lille, CNRS, UMR 8576 – UGSF – Unité de Glycobiologie Structurale et Fonctionnelle, 59000 Lille, France; 10 Service de Biologie (Unité de Microbiologie), Hôpital Mignot, Centre Hospitalier de Versailles, 177 rue de Versailles, 78150 Le Chesnay, France; 11 Service de Médecine Intensive Réanimation, Institut de Cardiologie, Assistance Publique–Hôpitaux de Paris (APHP), Sorbonne-Université, Hôpital Pitié–Salpêtrière, and Sorbonne Université, INSERM, UMRS_1166-ICAN Institute of Cardiometabolism and Nutrition, 47–83, Boulevard de l’Hôpital, 75651 Paris, France

## Abstract

**Background:**

Patients infected with the severe acute respiratory syndrome coronavirus 2 (SARS-COV 2) and requiring intensive care unit (ICU) have a high incidence of hospital-acquired infections; however, data regarding hospital acquired bloodstream infections (BSI) are scarce. We aimed to investigate risk factors and outcome of BSI in critically ill coronavirus infectious disease-19 (COVID-19) patients.

**Patients and methods:**

We performed an ancillary analysis of a multicenter prospective international cohort study (COVID-ICU study) that included 4010 COVID-19 ICU patients. For the present analysis, only those with data regarding primary outcome (death within 90 days from admission) or BSI status were included. Risk factors for BSI were analyzed using Fine and Gray competing risk model. Then, for outcome comparison, 537 BSI-patients were matched with 537 controls using propensity score matching.

**Results:**

Among 4010 included patients, 780 (19.5%) acquired a total of 1066 BSI (10.3 BSI per 1000 patients days at risk) of whom 92% were acquired in the ICU. Higher SAPS II, male gender, longer time from hospital to ICU admission and antiviral drug before admission were independently associated with an increased risk of BSI, and interestingly, this risk decreased over time. BSI was independently associated with a shorter time to death in the overall population (adjusted hazard ratio (aHR) 1.28, 95% CI 1.05–1.56) and, in the propensity score matched data set, patients with BSI had a higher mortality rate (39% vs 33% *p* = 0.036). BSI accounted for 3.6% of the death of the overall population.

**Conclusion:**

COVID-19 ICU patients have a high risk of BSI, especially early after ICU admission, risk that increases with severity but not with corticosteroids use. BSI is associated with an increased mortality rate.

**Supplementary Information:**

The online version contains supplementary material available at 10.1186/s13613-021-00971-w.

## Background

As a consequence of severe acute respiratory syndrome coronavirus-2 (SARS-COV 2) epidemic, intensive care units (ICU) worldwide faced a surge of critically ill patients who are at risk of developing bacterial infections, in particular patients requiring mechanical ventilation (MV) [1–3]. Although pulmonary bacterial infections (co-infection and nosocomial infections) have been extensively studied in ICU patients [1, 2, 4], conflicting results are reported, due to differences in infection definitions. Conversely, bloodstream infection (BSI) have been less studied, and among the 10 studies published to date [3, 5–12], only 2 focused on ICU patients [3, 9]. The first one was a single-center study that included 78 patients, and found a high incidence of BSI (45 episodes in 31 patients, e.g., 39% of patients with at least one episode) [9]. The second one, a case–cohort study that matched 235 ICU patients with coronavirus disease 2019 (COVID-19) to 235 patients without, found that COVID-19 patients had a higher rate of BSI than non-COVID-19 patients [3]. A third study included 100 BSI out of a cohort of 2005 patients, but although most bacteremia occurred in ICU patients, the baseline population was not exclusively hospitalized in the ICU [11]. Therefore, data regarding BSI (incidence, risk factors and prognosis) specifically in ICU patients are lacking.

We conducted this study to evaluate the incidence, risk factors and prognosis of hospital acquired BSI in patients with SARS-CoV-2 pneumonia hospitalized in the ICU.

## Methods

### Study design, patients

We performed an ancillary analysis of the COVID-ICU study. COVID-ICU was a multi-center, observational, and prospective cohort study conducted in 149 ICUs from 138 centers, across three countries (France, Switzerland, and Belgium) and has been described elsewhere [13]. It received approval from the ethical committee of the French Intensive Care Society (CE-SRLF 20–23) and Swiss and Belgium ethical committees following local regulations. All patients or close relatives were informed that their medical data were anonymously included in the COVID-ICU cohort. Patients and relatives had the possibility not to participate in the study. In case of refusal, the data were not collected accordingly. This manuscript follows the STROBE statement for reporting cohort studies.

For this report, we restricted the analysis to patients in whom the BSI status (yes/no) and day 90 status were known: these data were available for 4010 out of the 4747 patients included in the COVID-ICU study [13]. Data regarding incidence and risk factors were analyzed from this population. In a second set of analysis, to assess the attributable mortality of BSI, we matched 537 patients with BSI to 537 controls (patients without BSI) using a propensity score matching [14].

### Data collection

Day-1 was defined as the first day when the patient was in ICU at 10:00 AM. Each day, the study investigators completed a standardized electronic case report form. Baseline information collected at ICU admission were: age, gender, body mass index (BMI), active smoking, Simplified Acute Physiology Score (SAPS) II score [15], Sequential Organ Failure Assessment (SOFA) [16], comorbidities, immunodeficiency (if present), the date of the first symptom, dates and times of hospital and ICU admissions, and presence or not of co-infection at ICU admission [17]. Acute respiratory distress syndrome (ARDS) severity was assessed using Berlin definition [18]. Data collected daily from day 1 to day 15 and then at days 21, 45, 60 and 90 were the following: use of immunomodulatory drugs (interferon, tocilizumab or monoclonal antibodies), antiviral drug, antibiotics, anticoagulants and glucocorticoids; occurrence of BSI or ventilator-associated pneumonia (VAP); procedures during ICU stay (mechanical ventilation (MV), extracorporeal membrane oxygenation (ECMO), renal replacement therapy (RRT)). The number of days at risk for BSI was the number of days in hospital from the 48th hour of stay until ICU discharge for patients without BSI or until the first occurrence of BSI for patients with BSI.

For each positive blood culture, investigators could point out the micro-organisms responsible for infection among a restricted list: *Enterobacteriaceae*, *Pseudomonas aeruginosa*, *Acinetobacter baumannii*, *Streptococcus pneumonia*, Group A or B *Streptococcus*, *Enteroccoccus* spp., methicillin-susceptible *Staphylococcus aureus*, methicillin-resistant *Staphylococcus aureus*, *Haemophilus influenza*, anaerobes or other. Therefore, “other” denotes all micro-organisms not present in the preceding list and were not specified. Since some patients may have polymicrobial blood culture, investigators could declare as many micro-organisms that needed for a single blood sample.

The following outcomes were also recorded: occurrence of thrombosis [19], duration of MV, vital status at ICU and hospital discharge and 28, 60 and 90 days after ICU admission.

### Objectives and definition

Primary objective was to describe the incidence of bloodstream infection in patients hospitalized in the ICU for severe COVID-19 pneumonia. Secondary objectives were to describe risk factors for BSI, and to evaluate the attributable mortality of BSI.

Our study focused on hospital acquired BSI which was defined as a positive blood culture occurring ≥ 48 h after hospital admission, whereas ICU-acquired BSI was defined as a positive blood culture if it occurred ≥ 48 h after ICU admission. Therefore, patients were at risk of BSI from 48 h after hospital admission until hospital discharge, either dead or alive. Ventilator-free days at day 90 was defined as the number of days alive and breathing spontaneously (i.e., without mechanical ventilation) at day 90 after ICU admission [20]. ICU-free days at day 90 was defined as the number of days alive and outside the ICU at day 90 after ICU admission.

### Statistical analysis

Statistical analysis was performed with the statistical software R 3.4.3. Incidence rate and prevalence were expressed with the 95 percent confidence interval (95% CI). Categorical variables were expressed as number (percentage) and continuous variables as median and interquartile range [IQR]. When appropriate, the chi-square test and the Fisher’s exact test were used to compare categorical variables. The Mann–Whitney *U* test and the Wilcoxon test were used for continuous variables when applicable. All tests were two-sided, and a *P* value less than 0.05 was considered statistically significant.

Competitive risk analysis was used to estimate the probability of developing a BSI, with discharge and death being competing events. Using the “cmprsk” package we performed a Fine and Gray model to estimate sub-distribution hazard ratio (sdHR) of ICU death [14]. Therefore, sdHR > 1 indicates that those with exposure will be seen to have a quicker time to BSI. Conversely, a sdHR < 1 indicates a longer time before BSI onset for those exposed. A multivariable cox proportional hazard model was used for survival analysis. Variables associated with event (either BSI or death) with a *p* value < 0.2 in univariate analysis were included in multivariable model. Of note, for outcome comparison, only the first BSI was taken into account.

Because BSI acquisition was considered as a transition state from admission to discharge or death, patients with BSI were included in the group with BSI from first BSI onset only, to take into account immortal time bias associate with exposure.

To draw unbiased marginal estimates of exposure effect, a propensity-score matched analysis was performed. Propensity score was calculated for each patient and correspond to his probability to develop BSI and to die. As potential confounders, we included for propensity-score calculation all non-redundant variables associated with BSI (event) or death (outcome) with *p* value ≤ 0.05 in the Fine and Gray (BSI) or Cox model (death) multivariable analysis. Then, using the “MatchIt” package, a k-nearest neighbor algorithm was used for propensity-score matching with a 1:1 ratio: each patient with BSI was matched with 1 patient without BSI with the nearest propensity-score. The balance between matched groups was evaluated by the analysis of the standardized differences before and after weighting. A post-matching difference < 0.1 was considered as an optimal bias reduction. Multivariable Cox proportional hazard model and Kaplan–Meier survival curves were used for survival analysis in the propensity-matched analysis.

## Results

**Fig. 1:**
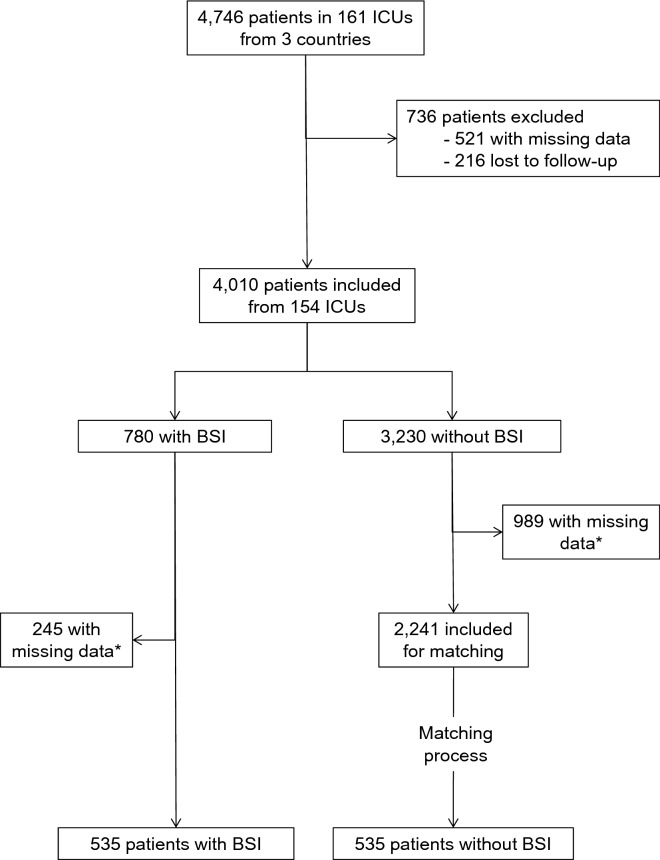
Flow chart of the study. ICU, intensive care unit. BSI, bloodstream infection. *1243 (989 in the no BSI group and 245 in the BSI group) patients had missing data among variables used for matching process and were, therefore, excluded from matched analysis

Among the 4747 patients included in the COVID-ICU database, 4010 had all data available and were included in the current analysis (Fig. 1). Their median [IQR] age was 62 [54–70] years, with admission SAPS II and SOFA scores of 37 [28–49] and 5 [3–8], respectively. ARDS criteria were objective in 3231 (81%), of whom 776 (19%), 1,635 (41%) and 820 had mild, moderate and severe ARDS, respectively. Finally 283 (7%) had a bacterial co-infection on admission.

**Fig. 2:**
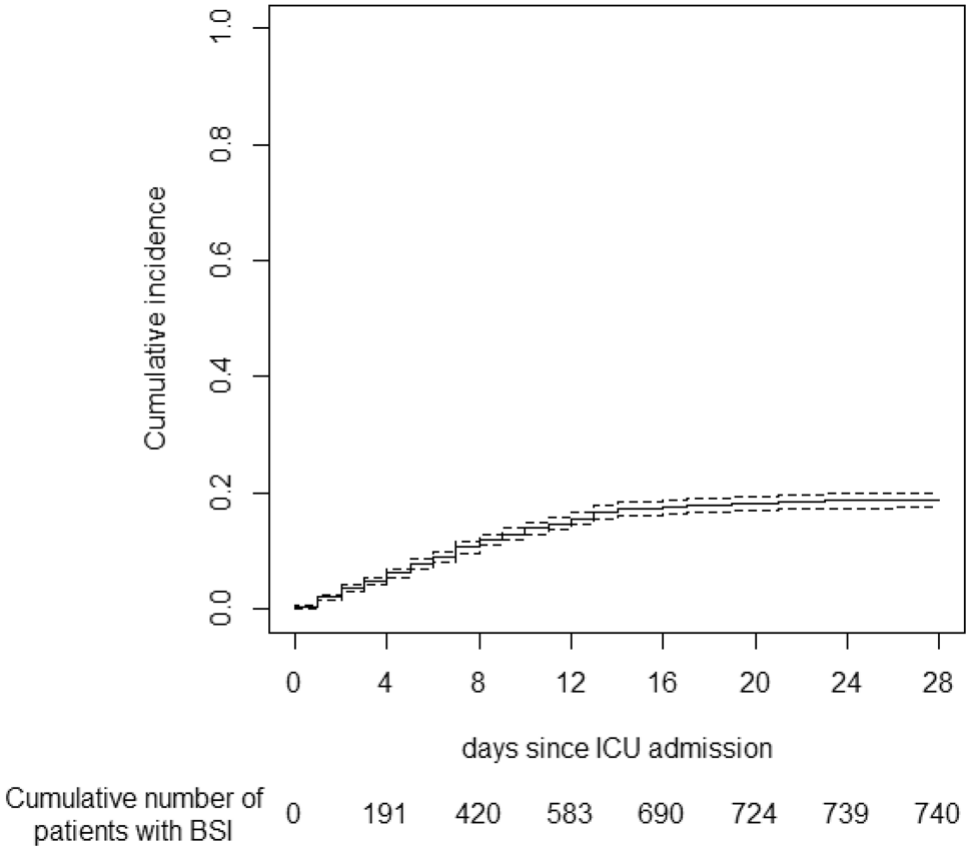
Cumulative incidence of bloodstream infection (BSI) as a function of time. ICU: intensive care unit. In 40 out of the 780 patients with BSI, BSI occurred after 21 days in the ICU. Precise day of BSI occurrence for these 40 patients is not known

Among these 4,010 patients, 780 (19.5%) experienced a total of 1066 episodes of BSI (1 [1, 2] episode per patient with BSI) through 103,293 patients-days at risk. Therefore, incidence rate was 10.3 BSI per 1000 patients-days. First episode of BSI occurred after a median [IQR] of 9 [5–13] days after hospital admission (Fig. 2). BSI was considered as ICU-acquired in 714 (92%) of patients with at least one BSI episode. Baseline characteristics of patients, according to their status (BSI or not) are displayed in Table 1. Briefly, patients experiencing BSI had higher disease severity on ICU admission, and were more likely to have severe lung disease, as assessed by the ARDS severity and the higher number of patients intubated upon admission. Micro-organisms responsible for infections are given in the Additional file 1: Table S1 (see Online Supplement). Of note, 104 (13%) of the 780 BSI patients relapsed (> 1 BSI episode with the same micro-organism), especially those infected with Methicillin-resistant *Staphylococcus aureus* (OR 4.2, 95% CI [1.4–12.8]; *p* = 0.012) or *Pseudomonas aeruginosa* (OR 2.9, 95% CI [1.2–6.8]; *p* = 0.017).

**Table 1:** Baseline characteristics of patients according to their status, namely, bloodstream infection or not

	Number with missing data	Patients with BSI *n* = 780	Patients without BSI *n* = 3230	*p*-value
Age, years	0	62 [53–69]	63 [54–71]	0.019
Frailty scale	395	2 [2, 3]	2 [2, 3]	0.80
Male gender	23	607 (78)	2346 (73)	0.004
Body mass index, kg/m^2^	292	29 [26–33]	28 [25–32]	0.006
Living place	69		3175	0.006
Admission from a long-term care facility		25 (3)	48 (2)	
Admission from nursing home		3 (0)	29 (1)	
Admission from home		738 (96)	3098 (3)	
Severity on admission				
SAPS II	323	40 [30–54]	36 [27–48]	< 0.001
SOFA score	562	7 [4–9]	4 [3–8]	< 0.001
ARDS severity on admission	820			< 0.001
No ARDS		54 (7)	365 (13)	
Mild ARDS		146 (19)	630 (22)	
Moderate ARDS		343 (46)	1292 (44)	
Severe ARDS		190 (26)	630 (22)	
Comorbidities				
No comorbidities	27	126 (16)	588 (18)	0.18
Alcohol consumption	827	26 (4)	134 (5)	0.28
Tabaco consumption	839	32 (5)	135 (5)	0.89
Chronic respiratory disease	677	183 (28)	671 (26)	0.23
Chronic heart failure	6	20 (3)	131 (4)	0.064
Hypertension	4	379 (49)	1523 (47)	0.47
Coronary artery disease	1	82 (11)	348 (11)	0.89
Diabetes mellitus	2	231 (30)	866 (27)	0.12
Hematological malignancy	0	22 (3)	87 (3)	0.94
Immunodepression	773	62 (10)	221 (9)	0.45
Solid malignancy	0	9 (1)	50(2)	0.51
Transplantation	0	21 (3)	62 (2)	0.22
Chronic renal failure	773	77 (24)	301 (23)	0.90
Cirrhosis	668	3 (0)	25 (1)	0.32
Neuromuscular disease	774	28 (4)	69 (3)	0.037
Home treatment				
Long-term corticosteroids treatment	1	36 (5)	129 (4)	0.49
Immunomodulatory drugs	1	42 (5)	125 (4)	0.072
Treatment with NSAID	536	58 (9)	171 (6)	0.022
Time from hospital admission to ICU, days	153	1 [0–3]	1 [0–3]	0.49
Period of admission	326			0.94
Before 15th of March 2020		46 (6)	187 (6)	
From 15th March to 31th of March 2020		439 (61)	1766 (60)	
From 1st April to 15 April 2020		193 (27)	841 (28)	
After 15th of April 2020		40 (5)	172 (5)	
Nurse/patient ratio	713	2 [2, 3]	2 [2, 3]	0.61
Admission during night-hours*	0	393 (50)	1570 (48)	0.39
Fever before admission	146	634 (85)	2567 (82)	0.16
Abdominal symptoms before admission	140	211 (27)	901 (28)	0.67
Co-infection at admission	123	76 (10)	247 (7.9)	0.070
Bacterial co-infection		66 (8)	217 (7)	0.10
Viral co-infection		4 (0)	21 (0)	0.86
Hospital/ICU treatment				
Antiviral treatment before admission	13	384 (49)	1451 (45)	0.033
Immunomodulatory drugs		12 (2)	47 (1)	0.99
Tocilizumab use		5 (1)	26 (1)	0.81
Intubation before admission	26	243 (31)	629 (20)	< 0.001
Management during period at risk for BSI				
Number of day at risk for BSI, days	143	7 [3–11]	12 [15–22]	< 0.001
ICU-acquired pneumonia during period at risk for BSI	165	238 (31)	1096 (34)	0.075
Corticosteroids during period at risk for BSI	21	171 (22)	969 (30)	< 0.001
Renal replacement therapy during period at risk for BSI	14	18 (2)	569 (18)	< 0.001
ECMO support during period at risk for BSI	14	68 (9)	195 (6)	0.009
Antibiotics use during period at risk for BSI	7	618 (79)	2871 (89)	< 0.001

Categorical variables are expressed as n (%) and continuous variables as median [interquartile range]

BSI: Bloodstream infection; SOFA: sequential-organ failure assessment; ARDS: Acute respiratory distress syndrome; NSAID: Non-steroidal anti-inflammatory drugs; ICU: Intensive care unit; RRT: renal replacement therapy; ECMO: Extra corporeal membrane oxygenation

*Admission during night hours was arbitrarily defined as admission in between 8:00 PM to 8:00 AM

### Risk factors for BSI

Risk factors for BSI in univariate and multivariable analysis are given in Table 2. Higher SAPS II, male gender, longer time from hospital to ICU admission, antiviral drug before admission, intubation during period at risk for BSI, renal replacement therapy during period at risk for BSI, antibiotic use prior to BSI were independently associated with occurrence of BSI and interestingly, the risk to develop BSI decreased over time (Table 2 and Fig. 2).

**Table 2:** Risk factors for bloodstream infection, using a Fine and Gray competing risk analysis

	Univariate analysis			Multivariable analysis		
	sdHR	95% CI	*p*-value	sdHR	95% CI	*p*-value
Age, per supplementary year	0.99	0.99–1.00	0.16	0.99	0.98–1.00	0.084
SAPS II score, per one point increment	1.01	1.01–1.01	< 0.001	1.01	1.00–1.02	0.046
SOFA at admission, per supplementary point	1.05	1.03–1.07	< 0.001	0.01	0.98–1.05	0.48
Mild ARDS	0.93	0.78–1.11	0.41			
Moderate ARDS	1.06	0.93–1.23	0.38			
Severe ARDS	1.21	1.02–1.42	0.025	1.00	0.77–1.30	0.99
Frailty scale, per supplementary point	0.98	0.92–1.05	0.61			
Male	1.23	1.04–1.46	0.013	1.41	1.08–1.84	0.011
BMI, per supplementary point	1.01	1.00–1.02	0.017	1.01	0.99–1.03	0.13
No comorbidities	0.99	0.83–1.19	0.96			
Tabaco consumption	0.94	0.65–1.35	0.72			
Chronic respiratory disease	1.2	1.01–1.42	0.037	1.01	0.79–1.28	0.96
Chronic heart failure	0.81	0.55–1.19	0.27			
Hypertension	1.02	0.88–1.17	0.83			
Coronary artery disease	1.04	0.83–1.29	0.76			
Diabetes mellitus	1.06	0.91–1.24	0.44			
Hematological malignancy	1.32	0.90–1.94	0.16	1.50	0.86–2.63	0.15
Immunodepression	1.02	0.78–1.34	0.89			
Solid malignancy	0.67	0.34–1.33	0.25			
Solid organ transplantation	1.08	0.67–1.75	0.74			
Chronic renal failure	0.89	0.74–1.07	0.23			
Cirrhosis	0.87	0.37–2.06	0.75			
Neuromuscular disease	1.5	1.02–2.21	0.042	1.29	0.73–2.30	0.38
Long-term corticosteroids treatment	1.07	0.76–1.5	0.69			
Immunomodulatory drugs	1.12	0.80–1.56	0.52			
Treatment with NSAID	1.18	0.88–1.58	0.27			
Admission from a long-term care facility	1.99	1.32–3.02	0.001	2.78	0.80–9.55	0.11
Admission from nursing home	0.61	0.23–1.59	0.31			
Admission from Home	0.75	0.55–1.03	0.072	2.34	0.93–5.85	0.070
Time from hospital admission to ICU, per supplementary day	1.01	0.99–1.03	0.2	1.03	1.02–1.05	< 0.001
Period of admission						
Before 15th of March 2020	0.94	0.70–1.28	0.71			
From 15th March to 31th of March 2020	1.07	0.93–1.23	0.34			
From 1st April to 15 April 2020	0.9	0.76–1.06	0.21			
After 15th of April 2020	1.01	0.74–1.39	0.93			
Nurse/patient ratio	0.93	0.84–1.04	0.20	0.91	0.78–1.06	0.23
Admission during night hours*	1.01	0.88–1.16	0.86			
Fever before admission	1.17	0.96–1.43	0.12	0.95	0.71–1.27	0.72
Abdominal symptoms before admission	0.90	0.77–1.06	0.20	1.19	0.93–1.51	0.17
Co-infection at admission	1.28	1.02–1.61	0.032	1.47	1.07–2.02	0.17
Bacterial co-infection	1.25	0.97–1.59	0.08			
Viral co-infection	0.14	0.03–0.78	0.025			
ICU acquired pneumonia during period at risk for BSI	0.84	0.73–0.98	0.025			
Antiviral treatment before admission	1.12	0.98–1.29	0.11	1.41	1.11–1.79	0.005
Immunomodulatory drugs during period at risk for BSI	1.14	0.66–1.97	0.64			
Tocilizumab during period at risk for BSI	0.8	0.33–1.92	0.62			
Intubation during period at risk for BSI	2.28	1.89–2.74	< 0.001	5.18	3.45–7.77	< 0.001
ECMO during period at risk for BSI	1.21	0.94–1.55	0.15	1.26	0.82–1.93	0.30
Thrombosis during period at risk for BSI	0.76	0.59–0.98	0.037	0.85	0.58–1.26	0.42
Renal replacement therapy during period at risk for BSI	0.29	0.22–0.40	< 0.001	0.30	0.18–0.49	< 0.001
Antibiotic during period at risk for BSI	0.52	0.43–0.62	< 0.001	0.45	0.33–0.61	< 0.001
Corticosteroids during period at risk for BSI	0.74	0.63–0.87	< 0.001	0.79	0.62–1.01	0.063
Number of day at risk for BSI, per supplementary days	0.97	0.96–0.98	< 0.001	0.93	0.92–0.95	< 0.001

sdHR: sub distribution hazard ratio; BSI: bloodstream infection; SAPS: simplified acute physiology score; SOFA: sequential-organ failure assessment; BMI: body mass index; ARDS: acute respiratory distress syndrome; NSAID: non-steroid anti-inflammatory drugs; ICU: intensive care unit; ECMO: extra corporeal membrane oxygenation

* Admission during night hours was arbitrarily defined as admission in between 8:00 PM to 8:00 AM

### Risk factors for day-90 death

Risk factors associated with death at day 90 are reported in Table 3. After adjusting for potential confounders using Cox model multivariable analysis, BSI occurring during hospital stay remained associated with day-90 mortality (HR 1.28, 95% CI 1.05–1.56). In a sensitive analysis in which BSI patients in whom the micro-organism responsible for infection was notified as “others” were excluded (*n* = 434), BSI remained independently associated with a shorter time to death (aHR1.41, 95% CI 1.08–1.84).

**Table 3:** Risk factors for death for the whole population

	Univariate analysis			Multivariable analysis		
	HR	95% CI	*p*-value	HR	95% CI	*p*-value
Age, per supplementary year	1.04	1.03–1.05	< 0.001	1.04	1.03–1.05	< 0.001
Frailty scale	1.37	1.31–1.42	< 0.001	1.33	1.24–1.42	< 0.001
Male	1.11	0.98–1.26	0.096	1.15	0.95–1.39	0.16
BMI > 25	0.82	0.73–0.92	< 0.001	0.83	0.69–1.01	0.056
SOFA at admission, per supplementary point	1.10	1.09–1.12	< 0.001	1.08	1.06–1.11	< 0.001
ARDS severity at admission						
No ARDS	Ref	Ref	Ref	Ref	Ref	Ref
Mild ARDS	1.07	0.84–1.35	0.60	1.02	0.73–1.43	0.90
Moderate ARDS	1.49	1.21–1.84	< 0.001	1.09	0.80–1.47	0.59
Severe ARDS	2.10	1.69–2.61	< 0.001	1.87	1.37–2.57	< 0.001
Comorbidities						
Alcohol consumption	1.15	0.89–1.49	0.27			
Tabaco consumption	1.05	0.81–1.37	0.70			
Chronic respiratory disease	1.10	0.96–1.25	0.17	0.90	0.74–1.08	0.26
Chronic heart failure	1.80	1.42–2.78	< 0.001	1.38	0.98–1.94	0.063
Coronary artery disease	1.78	1.54–2.06	< 0.001	1.16	0.92–1.46	0.21
Diabetes mellitus	1.51	1.35–1.69	< 0.001	1.21	1.02–1.43	0.033
Hematological malignancy	1.71	1.31–2.25	< 0.001	0.99	0.66–1.49	0.97
Immunodepression	1.44	1.20–1.73	< 0.001	1.12	0.75–1.69	0.58
Solid malignancy	1.95	1.36–2.78	< 0.001	1.09	0.58–2.02	0.79
Solid organ transplantation	1.98	1.48–2.66	< 0.001	1.09	0.56–2.12	0.80
Chronic renal failure	1.41	1.26–1.58	< 0.001	1.05	0.82–1.34	0.70
Cirrhosis	1.01	0.61–1.99	0.75			
Neuromuscular disease	0.94	0.66–1.32	0.71			
Usual medication						
Long term corticosteroids treatment	1.68	1.34–2.10	< 0.001	1.00	0.63–1.58	0.99
Immunomodulatory drugs	1.56	1.24–1.96	< 0.001	1.04	0.62–1.74	0.90
Treatment with NSAID	0.99	0.79–1.25	0.95			
Time from hospital admission to ICU, per supplementary day	1.00	0.99–1.01	0.92			
Period of admission						
Before 15th of March	Ref	Ref	Ref	Ref	Ref	Ref
From 15th March to 31th of March	0.74	0.60–0.91	0.004	0.92	0.67–1.25	0.58
From 1st April to 15 April	0.65	0.52–0.81	< 0.001	0.86	0.61–1.21	0.39
After 15th of April	0.50	0.35–0.68	< 0.001	0.71	0.44–1.14	0.15
Nurse/patient ratio	0.86	0.80–0.93	< 0.001	0.89	0.80–0.99	0.037
Admission during night hours*	0.98	0.88–1.09	0.68			
Fever before admission	0.84	0.73–0.96	0.013	0.85	0.69–1.04	0.11
Abdominal symptoms before admission	0.81	0.71–0.91	< 0.001	0.90	0.74–1.08	0.26
Co-infection at admission	1.25	1.05–1.51	0.015	1.06	0.80–1.40	0.68
Bacterial co-infection	1.28	1.06–1.55	0.012			
Viral co-infection	3.82	1.22–11.91	0.021			
ICU acquired pneumonia during period at risk for BSI^†^	1.10	0.98–1.23	0.093			
Antiviral treatment before admission	1.02	0.91–1.13	0.78			
Immunomodulatory drugs before admission	0.92	0.59–1.45	0.73			
Tocilizumab before admission	0.82	0.42–1.57	0.54			
Intubation before admission	1.32	1.17–1.49	< 0.001	0.88	0.71–1.09	0.25
Number of day at risk for BSI, per supplementary days	0.99	0.99–1.01	0.52			
Bloodstream infection	1.31	1.15–1.48	< 0.001	1.28	1.05–1.56	0.015

HR: hazard ratio; BSI: bloodstream infection; SOFA: sequential organ failure assessment; ARDS: acute respiratory distress syndrome; NSAID: non-steroid anti-inflammatory drugs; ICU: intensive care unit; ECMO: extracorporeal membrane oxygenation

* Admission during night hours was arbitrarily defined as admission in between 8:00 PM to 8:00 AM

^†^ Since ICU acquired pneumonia did not respect proportional assumption, the cox multivariable model was stratified on this variable

Univariate and multivariable Cox analysis of factors associated with day-90 death in the 780 patients with BSI is shown in Additional file 1: Table S3. Age, frailty scale [21], SOFA score the day of BSI, co-infection at admission, antibiotic and tocilizumab use during period at risk for BSI were associated with increased risk of day-90 death.

### Propensity score matched analysis

None redundant baseline characteristics independently associated with day-90 death and/or BSI (i.e., SAPS II, frailty scale, ARDS severity, hypertension, diabetes mellitus, male gender, time from hospital admission to ICU admission, antiviral before admission, intubation before admission, nurse/patients ratio) were included for propensity score calculation. Only patients without missing data regarding these variables were included for matching procedure (Fig. 1). Thereafter 537 patients with BSI were matched with 537 without BSI. Density of propensity scores in each groups are reported in Additional file 1: Fig. S1, and baseline characteristics were well balanced (Additional file 1: Table S2).

**Fig. 3:**
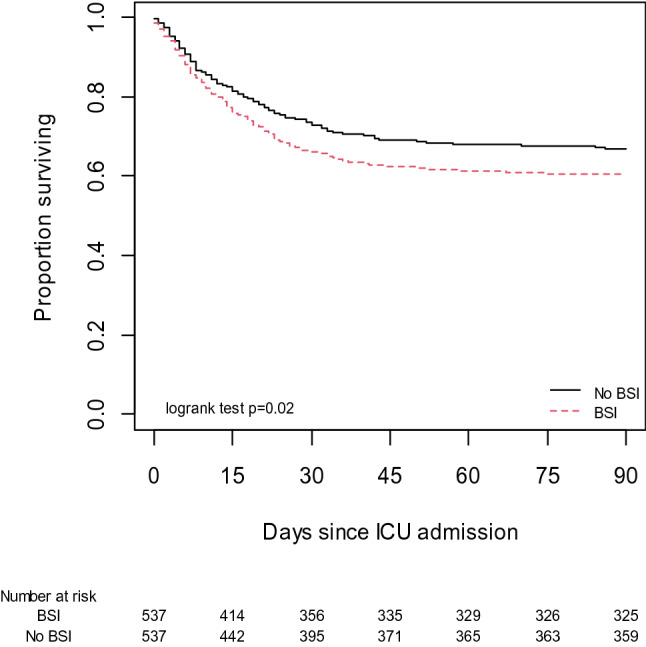
Kaplan–Meier analysis of survival in the matched patients with (*n* = 537) and without (*n* = 537) bloodstream infection (BSI). ICU: intensive care unit

Outcomes of matched patients with and without BSI are given in Table 4: patients with BSI had worse outcomes than patients without BSI with longer hospital and ICU length of stay, less day-90 ICU- and ventilator-free days, and higher mortality rate. Probability of death with time was higher among patients with BSI (HR 1.26; 95% CI [1.03–1.54]) (Fig. 3). Relative risk of death was 1.19 and number needed to harm was 16. Therefore, attributable mortality fraction of BSI in the overall population (*n* = 4010) was 3.6%.

**Table 4:** Outcomes among 537 patients with bloodstream infection and their 537 propensity-matched patients without bloodstream infection

	Patients with BSI *n *= 537	Patients without BSI *n* = 537	*p*-value
Length of stay in ICU, days	24 [15–36]	13 [6–25]	< 0.001
Length of stay in hospital, days	40 [27–58]	24 [13–40]	< 0.001
ICU-free days at day 90, days	52 [0–74]	65 [0–81]	< 0.001
Ventilator-free days at day 90, days	61 [0–81]	70 [0–88]	< 0.001
Death at day 90	212 (39)	178 (33)	0.036

Data are expressed as median [interquartile range] or *n* (%)

BSI: bloodstream infection; ICU: intensive care unit

## Discussion

In this study, we show that among a large population of COVID-19 patients requiring ICU, BSI was frequent, occurring in 19.5% of patients. Various risk factors for BSI were identified, with higher SAPS II, male gender, longer time from hospital to ICU admission, antiviral drug before admission, intubation being associated with increased risk of BSI. Another result was that BSI was independently associated with increased day-90 mortality.

Several studies having evaluated BSI in COVID-19 patients have been published to date, most of them mixing ICU and non-ICU patients, with incidence of BSI ranging from 2.7 to 5.6% [5, 6, 8, 10, 11]. Only 2 studies focused on ICU patients: a single-center study found that 31 out of 78 patients (39%) experienced at least one episode of BSI [9]; and a larger multicenter study of 235 COVID-19 patients found a 14.9% incidence of BSI [3]. In this study, the authors matched their COVID-19 patients to 235 ICU patients without COVID-19, and they found a lower rate of BSI in patients without COVID (3.4%). However, they did not evaluate the mortality attributable to BSI. A more recent multicenter case–control study matched, among 2,005 patients with COVID-19, 100 patients with BSI to 100 patients without BSI (matched on age, gender, and severity) [11]. The authors found that immunomodulatory drugs were not associated with an increased risk of BSI, but that BSI was associated with a higher mortality risk. However, this study did not focus on ICU patients. Our results are line with these data and complete them: our incidence of bacteremia was close to that of the largest ICU study published to date [3], and we showed for the first time that ICU-acquired BSI was associate with an excess death rate. This attributable mortality was in line with previous reports in non COVID-19 patients, ranking from 2.1 to 5.2% [14, 22]. Further studies are needed to better understand association between BSI and death in critically ill patients.

Given the high number of patients included in the COVID-ICU study and the high number of BSI, we were able to explore risk factors for BSI in ICU patients. Beyond traditional risk factors, unexpected results were observed for two variables: renal replacement therapy and number of day at risk being associated with lower risk of developing BSI. For the first variable, we assume that patients needing renal replacement therapy have a high probability of early death, competing with BSI occurrence. Similarly, use of epinephrine was not associated with an increased risk of BSI. For the second one, it suggests that patients have a higher risk of BSI soon after admission, while facing a more severe condition, whereas this risk decreases over time, with clinical improvement. Caregivers should be careful about BSI early after admission. Interestingly, corticosteroids use was not associated with increased risk of BSI. In a recent multicenter observational study, use of dexamethasone was not associated with an increased risk of BSI [23]. Similar results were found in another case–control study [11]. Data regarding immunomodulatory drug use and risk of BSI are discordant in ICU patients: a recent randomized placebo-controlled trial found no increased risk of infection with tocilizumab use [24]. Abelenda-Alonso et al. found no association between tocilizumab use and BSI [11], whereas Buetti et al. found an association between its use and BSI; however, in that study, the small sample size limits any firm conclusion [3]. In our study, although few patients received tocilizumab, its use was not associated with an increased risk of BSI. However, those experiencing BSI after tocilizumab use had an increased mortality as compared with BSI patients who did not received this agent. Combined with the lack of effect of anti-IL6 drugs in the most severe patients, their use should be cautiously outweighed in ICU patients. Similarly, BSI patients with previous antibiotic exposure had an increased risk of death. We hypothesized that antibiotic pressure selected resistant microorganisms in which empiric therapy was more likely to fail. This result supports a strict antibiotic stewardship program in COVID-19 setting.

Several limitations of our study should be underlined. First, some data are missing, which is inherent to this kind of multicenter observational study [13]. In particular, the main objective of the COVID-ICU study was not to evaluate infectious complications of ICU patients suffering from COVID-19. Therefore, we lack of important data, such as pathogen responsible for BSI (the list of bacteria available was restricted and for more than 50% of BSI episodes, pathogen responsible for infection is not known). Other relevant missing values were date of BSI occurrence after day 21 (for 40 patients, BSI occurred after day 21 but without additional precision), susceptibility of pathogen responsible for infection (wild type, multi-drug resistant…) appropriateness and duration of antimicrobial treatment. Second, this study was performed during the first wave, between March and May 2020. Since care of COVID-19 ICU patients has changed with improvement over time, and differ from one country to another, results might be different in different countries and among different waves of the pandemic. In particular, with the widespread use of corticosteroids and the increased use of anti-IL-6 drugs, incidence of BSI and outcomes may be different. Third, we defined BSI as a single positive blood culture. For some pathogens such as *Staphylococcus epidermidis*, 2 blood cultures taken apart are required to define BSI. We, therefore, might have overestimated the rate of BSI. However, the rate of BSI in our study was similar to other reports [3, 11] and lower than other [9]. Moreover, other studies found a high rate of BSI due to *Staphylococcus epidermidis* [12]. In our study, BSI patients infected with unknown pathogen and those infected with an identified micro-organism had similar outcomes, and should be both similarly considered. Fourth, as BSI occurred during ICU stay, we should acknowledge immortal time bias [25]. However, this bias would lead to underestimation of mortality risk associated with BSI, since patients experiencing this event are those surviving longer enough, whereas those who died early have short time of exposition but were even included in control group.

In conclusion, BSI is a frequent complication of critically ill COVID-19 patients, especially early after ICU admission and is associated with increased severity at admission but not with corticosteroids use. BSI is associated with an increased mortality rate.

## Supplementary Information

**Additional file 1.** Supplementary results.

## Data Availability

The data sets generated during the current study are available from the corresponding author on reasonable request.

## References

[1] Luyt C-E, Sahnoun T, Gautier M, Vidal P, Burrel S, Pineton de Chambrun M (2020). Ventilator-associated pneumonia in patients with SARS-CoV-2-associated acute respiratory distress syndrome requiring ECMO: a retrospective cohort study. Ann Intensive Care.

[2] Rouzé A, Martin-Loeches I, Povoa P, Makris D, Artigas A, Bouchereau M (2021). Relationship between SARS-CoV-2 infection and the incidence of ventilator-associated lower respiratory tract infections: a European multicenter cohort study. Intensive Care Med.

[3] Buetti N, Ruckly S, de Montmollin E, Reignier J, Terzi N, Cohen Y (2021). COVID-19 increased the risk of ICU-acquired bloodstream infections: a case-cohort study from the multicentric OUTCOMEREA network. Intensive Care Med.

[4] Razazi K, Arrestier R, Haudebourg AF, Benelli B, Carteaux G, Decousser J-W (2020). Risks of ventilator-associated pneumonia and invasive pulmonary aspergillosis in patients with viral acute respiratory distress syndrome related or not to Coronavirus 19 disease. Crit Care.

[5] Goyal P, Choi JJ, Pinheiro LC, Schenck EJ, Chen R, Jabri A (2020). Clinical characteristics of Covid-19 in New York City. N Engl J Med.

[6] Hughes S, Troise O, Donaldson H, Mughal N, Moore LSP (2020). Bacterial and fungal coinfection among hospitalized patients with COVID-19: a retrospective cohort study in a UK secondary-care setting. Clin Microbiol Infect.

[7] Sepulveda J, Westblade LF, Whittier S, Satlin MJ, Greendyke WG, Aaron JG (2020). Bacteremia and blood culture utilization during COVID-19 surge in New York City. J Clin Microbiol.

[8] Engsbro AL, Israelsen SB, Pedersen M, Tingsgaard S, Lisby G, Andersen CØ (2020). Predominance of hospital-acquired bloodstream infection in patients with Covid-19 pneumonia. Infect Dis.

[9] Giacobbe DR, Battaglini D, Ball L, Brunetti I, Bruzzone B, Codda G (2020). Bloodstream infections in critically ill patients with COVID-19. Eur J Clin Invest.

[10] Søgaard KK, Baettig V, Osthoff M, Marsch S, Leuzinger K, Schweitzer M (2021). Community-acquired and hospital-acquired respiratory tract infection and bloodstream infection in patients hospitalized with COVID-19 pneumonia. J Intensive Care.

[11] Abelenda-Alonso G, Rombauts A, Gudiol C, Oriol I, Simonetti A, Coloma A (2021). Immunomodulatory therapy, risk factors and outcomes of hospital-acquired bloodstream infection in patients with severe COVID-19 pneumonia: a Spanish case-control matched multicenter study (BACTCOVID). Clin Microbiol Infect.

[12] Bhatt PJ, Shiau S, Brunetti L, Xie Y, Solanki K, Khalid S (2021). Risk factors and outcomes of hospitalized patients with severe Coronavirus Disease 2019 (COVID-19) and secondary bloodstream infections: a multicenter case-control study. Clin Infect Dis.

[13] COVID-ICU Group on behalf of the REVA Network and the COVID-ICU Investigators. Clinical characteristics and day-90 outcomes of 4244 critically ill adults with COVID-19: a prospective cohort study. Intensive Care Med. 2021;47:60–73. 10.1007/s00134-020-06294-x PMC7674575 33211135

[14] Massart N, Wattecamps G, Moriconi M, Fillatre P (2021). Attributable mortality of ICU acquired bloodstream infections: a propensity-score matched analysis. Eur J Clin Microbiol Infect Dis.

[15] Le Gall JR, Lemeshow S, Saulnier F (1993). A new Simplified Acute Physiology Score (SAPS II) based on a European/North American multicenter study. JAMA.

[16] Vincent JL, Moreno R, Takala J, Willatts S, De Mendonça A, Bruining H (1996). The SOFA (Sepsis-related Organ Failure Assessment) score to describe organ dysfunction/failure. On behalf of the Working Group on sepsis-related problems of the European Society of Intensive Care Medicine. Intensive Care Med.

[17] Dres M, Hajage D, Lebbah S, Kimmoun A, Pham T, Béduneau G (2021). Characteristics, management, and prognosis of elderly patients with COVID-19 admitted in the ICU during the first wave: insights from the COVID-ICU study: prognosis of COVID-19 elderly critically ill patients in the ICU. Ann Intensive Care.

[18] ARDS Definition Task Force, Ranieri VM, Rubenfeld GD, Thompson BT, Ferguson ND, Caldwell E, et al. Acute respiratory distress syndrome: the Berlin Definition. JAMA. 2012;307:2526–33. 10.1001/jama.2012.5669 22797452

[19] Hékimian G, Masi P, Lejeune M, Lebreton G, Chommeloux J, Desnos C (2021). Extracorporeal membrane oxygenation induces early alterations in coagulation and fibrinolysis profiles in COVID-19 patients with acute respiratory distress syndrome. Thromb Haemost.

[20] Papazian L, Jaber S, Hraiech S, Baumstarck K, Cayot-Constantin S, Aissaoui N (2021). Preemptive ganciclovir for mechanically ventilated patients with cytomegalovirus reactivation. Ann Intensive Care.

[21] Bruno RR, Wernly B, Flaatten H, Fjølner J, Artigas A, Bollen Pinto B (2021). Lactate is associated with mortality in very old intensive care patients suffering from COVID-19: results from an international observational study of 2860 patients. Ann Intensive Care.

[22] Prowle JR, Echeverri JE, Ligabo EV, Sherry N, Taori GC, Crozier TM (2011). Acquired bloodstream infection in the intensive care unit: incidence and attributable mortality. Crit Care.

[23] Gragueb-Chatti I, Lopez A, Hamidi D, Guervilly C, Loundou A, Daviet F (2021). Impact of dexamethasone on the incidence of ventilator-associated pneumonia and blood stream infections in COVID-19 patients requiring invasive mechanical ventilation: a multicenter retrospective study. Ann Intensive Care.

[24] Gupta S, Wang W, Hayek SS, Chan L, Mathews KS, Melamed ML (2021). Association between early treatment with tocilizumab and mortality among critically ill patients with COVID-19. JAMA Intern Med.

[25] Vail EA, Gershengorn HB, Wunsch H, Walkey AJ (2021). Attention to immortal time bias in critical care research. Am J Respir Crit Care Med.

