# Supplementary material for: Characteristics and prognosis of bloodstream infection in patients with COVID-19 admitted in the ICU: an ancillary study of the COVID-ICU study
Source: Ann Intensive Care. 2021 Dec 24;11:183. doi: 10.1186/s13613-021-00971-w (PMC8708508; doi:10.1186/s13613-021-00971-w)
Supplement: Supplementary file 1 — Additional file 1. Supplementary results. [file 13613_2021_971_MOESM1_ESM.docx]

**Characteristics and prognosis of bloodstream infection in patients with COVID-19 admitted in the ICU**

**An ancillary study of the COVID-ICU study**

N Massart, V Maxime, P Fillatre K Razazi, A Ferré, P Moine, F Legay, G Voiriot, M Amara, F Santi, S Nseir, S Marque-Juillet, R Bounab, N, Barbarot, F Bruneel, CE Luyt and the COVID-ICU Bacteremia Study Group on behalf of the COVID-ICU Investigators.

ONLINE SUPPLEMENT

**eTable1.** Pathogens responsible for bloodstream infections

| Micro-organism | Pathogens responsible for first BSI episode  N = 780* | Pathogens responsible for all BSI episodes  N = 1101* |
| --- | --- | --- |
| *Enterobacteriaceae* | 112 (14) | 146 (18) |
| *Staphylococcus aureus*  Methicillin-susceptible  Methicillin-resistant | 67 (9)  26 (3) | 85 (8)  37 (3) |
| *Pseudomonas aeruginosa* | 50 (6) | 69 (6) |
| *Enterococcus spp.* | 45 (6) | 57 (5) |
| *Streptococcus pneumoniae* | 8 (1) | 11 (1) |
| *Non pneumonia Streptococcus*† | 10 (1) | 68 (6) |
| Anaerobes | 18 (2) | 25 (2) |
| Other ‡ | 434 (56) | 593 (54) |
| Polymicrobial | 73 (9) | 55 (5) |
| Missing data | 10 (1) | 10 (1) |

Results are expressed as n (%)

Abbreviation: BSI, bloodstream infection

***** Number of pathogens is superior to number of episodes since some episodes of BSI are polymicrobial

†Except *Streptococcus pneumoniae*

‡ “Other” was considered in BSI due to *Haemophilus influen za* or *Acinetobacter baumannii or* when the micro-organisms responsible for infection was not mentioned in the dataset.

**eTable2.** Baseline characteristics of patients with BSI and propensity score matched controls.

|  | Patients without BSI  n = 537 | Patients with BSI  n = 537 | p-value | Mean difference |
| --- | --- | --- | --- | --- |
| Age, year | 61 [52, 69] | 61 [53, 69] | 0.876 | 0.34 |
| Frailty score | 2 [1, 3] | 2 [2, 3] | 0.809 | 0.01 |
| Male, – no. (%) | 420 (78.2) | 422 (78.6) | 0.941 | 0.004 |
| Body mass index, kg/m² | 29.38 [26.26, 33.63] | 29.09 [26.12, 33.63] | 0.671 | 0 |
| Living place |  |  | 0.231 |  |
| Admission from a long-term care facility– no. (%) | 4 (0.7) | 2 (0.4) |  | 0.003 |
| Admission from nursing home– no. (%) | 5 (0.9) | 11 (2.0) |  | 0.011 |
| Admission from Home – no. (%) | 528 (98.3) | 524 (97.6) |  | 0.007 |
| SOFA at admission | 6 [3, 10] | 7 [4, 10] | 0.128 | 0.25 |
| SAPS II at admission | 39 [29, 55] | 40 [30, 53] | 0.516 | 0.25 |
| ARDS severity at admission |  |  | 0.306 |  |
| No ARDS | 46 (8.6) | 44 (8.2) |  | 0.004 |
| Mild ARDS | 107 (19.9) | 100 (18.6) |  | 0.013 |
| Moderate ARDS | 224 (41.7) | 244 (45.4) |  | 0.037 |
| Severe ARDS | 152 (28.3) | 147 (27.4) |  | 0.009 |
| Comorbidities |  |  |  |  |
| No comorbidities | 108 (20.1) | 94 (17.5) | 0.317 | -0.026 |
| Alcohol consumption | 17 (4.0) | 21 (4.8) | 0.661 | 0.008 |
| Tobacco consumption | 20 (4.8) | 23 (5.3) | 0.827 | 0.005 |
| Chronic respiratory disease | 112 (26.4) | 121 (27.6) | 0.731 | 0.012 |
| Chronic heart failure | 21 (3.9) | 16 (3.0) | 0.503 | 0.009 |
| Hypertension | 259 (48.2) | 260 (48.4) | 1.000 | 0.002 |
| Coronary artery disease | 51 (9.5) | 51 (9.5) | 1.000 | 0 |
| Diabetes mellitus | 158 (29.4) | 158 (29.4) | 1.000 | 0 |
| Hematological malignancy | 12 (2.2) | 12 (2.2) | 1.000 | 0 |
| Immunodepression | 29 (6.8) | 43 (9.8) | 0.140 | 0.03 |
| Solid malignancy | 8 (1.5) | 7 (1.3) | 1.000 | 0.002 |
| Solid organ transplantation | 7 (1.3) | 15 (2.8) | 0.132 | 0.015 |
| Chronic renal failure | 57 (13.3) | 50 (11.4) | 0.432 | 0.019 |
| Cirrhosis | 2 (0.5) | 3 (0.7) | 1.000 | 0.002 |
| Neuromuscular disease | 13 (3.0) | 22 (5.0) | 0.199 | 0.020 |
| Usual medication |  |  |  |  |
| Long term corticosteroids treatment | 19 (3.5) | 25 (4.7) | 0.441 | 0.012 |
| Immunomodulatory drugs | 20 (3.7) | 26 (4.8) | 0.451 | 0.011 |
| Treatment with NSAID |  |  |  |  |
| Time from hospital admission to ICU, days | 1 [0, 3] | 1 [0, 3] | 0.938 | 0.09 |
| Period of admission |  |  | 0.779 |  |
| Before 15^th^ of March | 28 (5.6) | 30 (5.9) |  | 0.003 |
| From 15^th^ March to 31th of March | 304 (60.6) | 317 (62.5) |  | 0.019 |
| From 1^st^ April to 15 April | 141 (28.1) | 128 (25.2) |  | 0.029 |
| After 15^th^ of April | 29 (5.8) | 32 (6.3) |  | 0.005 |
| Nurse/patient ratio | 1.94 [1.68, 2.47] | 2.00 [1.68, 2.40] | 0.784 | 0.04 |
| Admission during night hours* | 279 (52.0) | 271 (50.5) | 0.669 | 0.015 |
| Fever before admission | 448 (85.7) | 441 (85.0) | 0.821 | 0.007 |
| Abdominal symptoms before admission | 129 (24.0) | 142 (26.4) | 0.399 | 0.024 |
| Co-infection at admission | 44 (8.3) | 59 (11.2) | 0.141 | 0.029 |
| Antiviral treatment before admission | 258 (48.0) | 266 (49.5) | 0.669 | 0.015 |
| Tocilizumab before admission | 7 (1.3) | 3 (0.6) | 0.341 | 0.007 |
| Corticosteroids during period at risk for BSI | 190 (35.4) | 121 (22.5) | <0.001 | 0.129 |
| Intubation before admission | 176 (32.8) | 176 (32.8) | 1.000 | 0 |

Categorical variables are expressed as n(%) and continuous variables as median (interquartile range).

Abbreviations. BSI : Bloodstream infection. SOFA : score of organ failure assessment. ARDS: Acute respiratory distress syndrome. NSAID : Non-steroidal anti-inflammatory drugs. ICU : Intensive care unit. ECMO : Extra corporeal membrane oxygenation.

* admission during night hours was arbitrarily defined as admission in between 8:00 PM to 8:00 AM

**eTable 3.** Univariate and multivariable Cox analysis of factors associated with day-90 death in the 780 patients with bloodstream infection

|  | Univariate analysis | | | Multivariate analysis | | |
| --- | --- | --- | --- | --- | --- | --- |
|  | HR | 95% CI | p-value | HR | 95% CI | p-value |
| Age, per supplementary year | 1.02 | 1.01-1.03 | <0.001 | 1.02 | 1.01-1.04 | <0.001 |
| Frailty score | 1.37 | 1.31-1.42 | <0.001 | 1.16 | 1.04-1.30 | 0.008 |
| Male | 0.99 | 0.75-1.29 | 0.924 |  |  |  |
| BMI >25 | 0.85 | 0.66-1.10 | 0.215 |  |  |  |
| SOFA score on the day of BSI, per supplementary point | 1.06 | 1.03-1.08 | <0.001 | 1.05 | 1.02-1.08 | <0.001 |
| ARDS severity at admission |  |  |  |  |  |  |
| No ARDS | Ref | Ref | Ref |  |  |  |
| Mild ARDS | 0.76 | 0.45-1.25 | 0.28 |  |  |  |
| Moderate ARDS | 1.0 | 0.64-1.57 | 0.994 |  |  |  |
| Severe ARDS | 1.22 | 0.77-1.95 | 0.398 |  |  |  |
| Comorbidities |  |  |  |  |  |  |
| Alcohol consumption | 0.96 | 0.53-1.76 | 0.903 |  |  |  |
| Tabaco consumption | 1.09 | 0.64-1.87 | 0.747 |  |  |  |
| Chronic respiratory disease | 0.983 | 0.0.75-1.28 | 0.902 |  |  |  |
| Chronic heart failure | 1.90 | 1.04-3.48 | 0.035 | 1.78 | 0.35-1.74 | 0.54 |
| Hypertension | 1.34 | 1.08-1.68 | 0.009 | 0.95 | 0.70-1.28 | 0.74 |
| Coronary artery disease | 1.87 | 1.37-2.56 | <0.001 | 1.32 | 0.89-1.96 | 0.17 |
| Diabetes | 1.33 | 1.06-1.69 | 0.015 | 1.27 | 0.94-1.71 | 0.12 |
| Hematological malignancy | 1.56 | 0.87-2.77 | 0.133 | 1.69 | 0.89-3.24 | 0.11 |
| Immunodepression | 1.23 | 0.85-1.81 | 0.276 |  |  |  |
| Solid malignancy | 1.27 | 0.47-3.40 | 0.635 |  |  |  |
| Solid organ transplantation | 1.59 | 0.89-2.84 | 0.114 | 0.89 | 0.34-2.27 | 0.80 |
| Chronic renal failure | 1.41 | 1.26-1.58 | <0.001 | 1.42 | 0.91-2.20 | 0.12 |
| Cirrhosis | 1.88 | 0.47-7.58 | 0.372 |  |  |  |
| Neuromuscular disease | 0.65 | 0.32-1.31 | 0.227 |  |  |  |
| Usual medication |  |  |  |  |  |  |
| Long term corticosteroids treatment | 1.49 | 0.95-2.34 | 0.084 | 0.85 | 0.38-1.90 | 0.74 |
| Immunomodulatory drugs | 1.68 | 1.11-2.55 | 0.014 | 1.15 | 0.50-2.68 | 0.70 |
| Treatment with NSAID | 0.96 | 0.62-1.49 | 0.857 |  |  |  |
| Time from hospital admission to ICU, per supplementary day | 0.99 | 0.97-1.02 | 0.629 |  |  |  |
| Period of admission |  |  |  |  |  |  |
| Before 15^th^ of March | Ref | Ref | Ref | Ref | Ref | Ref |
| From 15^th^ March to 31th of March | 0.58 | 0.39-0.88 | 0.009 | 0.49 | 0.31-0.77 | 0.002 |
| From 1^st^ April to 15 April | 0.55 | 0.35-0.85 | 0.008 | 0.44 | 0.26-0.74 | 0.002 |
| After 15^th^ of April | 0.39 | 0.20-0.78 | 0.007 | 0.26 | 0.12-0.54 | <0.001 |
| Nurse/patient ratio | 0.92 | 0.79-1.07 | 0.293 |  |  |  |
| Admission during night hours* | 0.95 | 0.76-1.19 | 0.66 |  |  |  |
| Fever before admission | 0.88 | 0.65-1.20 | 0.431 |  |  |  |
| Abdominal symptoms before admission | 0.92 | 0.71-1.18 | 0.498 |  |  |  |
| Co-infection at admission | 1.47 | 1.05-2.05 | 0.025 | 1.63 | 1.07-2.49 | 0.023 |
| Bacterial co infection | 1.38 | 0.96 -1.99 | 0.086 |  |  |  |
| Viral co infection | 3.82 | 1.22-11.91 | 0.021 |  |  |  |
| ICU acquired pneumonia during period at risk for BSI* | 0.89 | 0.70-1.14 | 0.349 |  |  |  |
| Antiviral treatment during period at risk for BSI | 1.15 | 0.92-1.43 | 0.226 |  |  |  |
| Tocilizumab during period at risk for BSI | 2.26 | 1.01-5.07 | 0.048 | 3.05 | 1.24-7.51 | 0.015 |
| Corticosteroids during period at risk for BSI | 0.96 | 0.76-1.21 | 0.75 |  |  |  |
| Antibiotic during period at risk for BSI | 1.40 | 1.04-1.88 | 0.026 | 1.58 | 1.07-2.35 | 0.023 |
| Intubation before admission | 0.81 | 0.63-1.03 | 0.085 | 0.75 | 0.55-1.04 | 0.082 |
| Number of day at risk for BSI, per supplementary days | 1.00 | 0.98-1.02 | 0.741 |  |  |  |
| Microbiology  Other micro-organism  Anaerobes  *Enterobacteriaceae*  *Enterococcus* spp.  *Streptococcus pneumoniae*  *Pseudomonas aeruginosa*  MSSA  MRSA  *Streptococcus* sp*.* | Ref  0.58  1.14  0.65  1.04  0.76  1.05  1.27  1.05 | Ref  0.24-1.40  0.83-1.55  0.37-1.14  0.33-3.25  0.45-1.26  0.71-1.56  0.73-2.24  0.39-2.82 | Ref  0.222  0.426  0.134  0.95  0.284  0.804  0.399  0.925 | Ref  0.66  1.03  0.83  1.76  0.57  1.05  2.08  1.89 | Ref  0.21-2.08  0.70-1.52  0.44-1.57  0.42-7.40  0.30-1.08  0.64-1.72  0.99-4.36  0.68-5.31 | Ref  0.48  0.86  0.57  0.44  0.087  0.86  0.052  0.22 |

Abbreviations. HR : hazard ratio. BSI: bloodstream infection. SOFA: sequential organ failure assessment. ARDS: acute respiratory distress syndrome. ICU : Intensive care unit. MSSA: methicillin-susceptible *Staphylococcus aureus*. MRSA: methicillin-resistant *Staphylococcus aureus*

**eFigure 1**. Density of propensity score in patients. A: All patients with bloodstream infection. B: matched patients with bloodstream infection. C: all patients without bloodstream infection. D: matched patients without bloodstream infection.
